# Comparative Genomic and Epidemiologic Analysis of Methicillin-Resistant *Staphylococcus aureus* Isolates in Republic of Korea

**DOI:** 10.3390/antibiotics15030235

**Published:** 2026-02-24

**Authors:** Dong-Hyun Kim, Du-Gyeong Han, Sungkyoung Lee, Jung-Sik Yoo, Se-Mi Jeon

**Affiliations:** Department of Bacterial Disease Research, Center for Infectious Disease Research, National Institute of Infectious Disease, Korea National Institute of Health, Korea Disease Control and Prevention Agency, Cheongju 28159, Republic of Korea; kgwkk2@gmail.com (D.-H.K.); ktf0222@korea.kr (D.-G.H.); serenity98@korea.kr (S.L.); jungsiku@korea.kr (J.-S.Y.)

**Keywords:** methicillin-resistant *Staphylococcus aureus*, epidemiology, whole-genome sequencing, antimicrobial resistance

## Abstract

**Background/Objectives**: Methicillin-resistant *Staphylococcus aureus* (MRSA) is a major causative pathogen in Republic of Korea. While numerous variants exist, the long-term evolutionary history of indigenous lineages remains unclear. Therefore, this study aimed to reconstruct the high-resolution population structure of Korean MRSA. **Methods**: A total of 191 MRSA clinical isolates collected between 1999 and 2025 were obtained from four Korean biobanks. Whole-genome sequencing was conducted and international MRSA genomes from the National Center for Bioinformatics were used as a control group. A genome-wide association study, including single-nucleotide polymorphism (SNP)-based phylogenomic analysis, principal component analysis (PCA), and ADMIXTURE, was performed for distribution analysis. A time-scale epidemiological analysis was conducted using SNP-based phylogenetic data. Additional profiling was performed via core genome multilocus sequence typing (cgMLST) for comparison with the SNP-based phylogenomic results. Finally, antimicrobial resistance and virulence factor genes were annotated using the ResFinder and VirulenceFinder databases. **Results**: Phylogenetic analysis identified five major clades: 1 (ST5), 2 (ST6), 3 (ST72), 4 (ST1/ST188), and 5 (ST8/ST239/ST254). Time-scaled analysis estimated that these major clades began to diverge in the early 20th century (e.g., Clade 1 around 1918). Notably, Korean ST5 isolates formed a sublineage distinct from North American strains, characterized by unique AMR profiles and divergence in the 1960s. ST72 formed an independent clade that was phylogenetically closer to clade 4 (ST1/ST188) than to the canonical CC8 group (clade 5). Furthermore, the ST1 isolates showed a temporal split into an older lineage and a recent sublineage, with expanded AMR pro-files. **Conclusions**: By integrating time-scale phylogenetics with cgMLST, we elucidated the evolutionary history and transmission dynamics of Korean MRSA.

## 1. Introduction

Methicillin-resistant *Staphylococcus aureus* (MRSA) is a major pathogen found in hospitals and communities in Republic of Korea. In the 2000s, MRSA accounted for 60–70% of all *S. aureus* isolates in Korean tertiary hospitals, with reported methicillin resistance rates as high as 81% in 2009 [1]. This endemic presence made MRSA a leading cause of nosocomial infections in Republic of Korea. However, over the past decade, concerted infection control efforts have led to a gradual decline in the incidence of MRSA. Nationwide surveillance indicates that oxacillin resistance among *S. aureus* has decreased from ~76% in 2008 to ~62.5% in 2016, reflecting a reduced outbreak frequency [2]. Even so, MRSA remains a critical threat. It continues to cause a large proportion (nearly half) of *S. aureus* bloodstream infections in Republic of Korea [3]. It is frequently implicated in device-associated infections, such as prosthetic joint- and catheter-related infections. In particular, *S. aureus* (including methicillin-resistant *Staphylococcus aureus* [MRSA]) is the most common cause of osteoarticular infections, with MRSA representing <40% of staphylococcal bone/joint cases reported in Korean studies [4]. These data underscore that, while overall MRSA rates have improved, vulnerable settings, such as implanted medical devices and surgical sites, remain at risk of MRSA infection.

A feature of MRSA is its genetic heterogeneity; the pathogen comprises numerous lineages (or clones) with distinct antibiotic resistance profiles and virulence factors. In Republic of Korea, epidemiological surveys conducted from 2001 to 2008 identified nine major MRSA clones (designated KMRSA-1 to -9) based on multilocus genotypes and molecular characteristics. This diversity makes precise strain classification essential. By delineating the clone to which an MRSA isolate belongs, clinicians and epidemiologists can better predict its resistance patterns and track its spread. Public health monitoring relies on clone typing to identify outbreaks and transmission trends [1]. Traditional typing methods such as pulsed-field gel electrophoresis (PFGE) are good standards for local outbreak investigations but are less suitable for long-term comparisons [5]. Currently, the most widely used approach for broader MRSA classification is multilocus sequence typing (MLST), which sequences seven housekeeping genes of a bacterium to assign an allelic profile or sequence type (ST). STs that share ≥ 5 of 7 MLST alleles are grouped into a clonal complex (CC), representing a broader lineage [5].

Molecular epidemiological studies have consistently shown that two MRSA lineages have predominated Republic of Korea in recent years: ST72 and Type IV. It was first recognized in Korean community infections in the mid-2000s and quickly became the dominant clone in both community and hospital settings [6,7]. ST72 MRSA has essentially become endemic in Korean healthcare: even hospital-associated MRSA bacteremias are frequently caused by this ST. By contrast, ST5 (CC5) is a classic hospital-associated MRSA lineage (often carrying SCC*mec* II) that has been widespread in hospitals worldwide (known as the “New York/Japan” clone or related to EMRSA-3). In Republic of Korea, ST5-SCC*mec* II is the leading clone responsible for nosocomial infections and is highly prevalent in certain settings. For example, a pediatric study found that 67.6% of MRSA bacteremia cases from 2016 to 2021 were caused by ST72-group strains and ~18.9% were caused by ST5-group strains, accounting for ~86% of cases [7]. Although MLST provides a convenient high-level classification, it lacks the genomic resolution to reconstruct precise transmission histories or evolutionary timescales across eras. Major STs, such as ST1, ST5, ST8, and ST72, have circulated for decades and accumulated many genomic changes without altering their MLST type. Consequently, while major clones have been identified, the temporal evolutionary dynamics of dominant MRSA lineages in Republic of Korea remain insufficiently resolved. For instance, one analysis found that within the dominant ST72 clone, WGS could partition isolates into multiple clades with distinct resistance and virulence gene profiles, insights impossible to obtain from MLST since all were simply “ST72”.

Thus, isolates can be epidemiologically distinct yet appear identical by MLST. This lack of resolution poses a significant challenge for infection prevention and control (IPC). Understanding the precise evolutionary dynamics and temporal divergence of these lineages is not merely of academic interest; it is critical for patient care. Detecting the emergence of sub-lineages with enhanced fitness or expanded resistance profiles allows for more timely interventions and informs empiric antibiotic stewardship strategies to mitigate the spread of high-risk clones in healthcare settings.

Recently, whole-genome phylogenetic analyses have been broadly utilized to study MRSA evolution and transmission at high resolution. Whole-genome single-nucleotide poly (SNP) analysis, which compares single-nucleotide polymorphisms across the core genome, facilitates the construction of precise phylogenetic trees. Time-scaled phylogenomic analyses can be performed by applying molecular clock models to the SNP data. Such approaches are exceptionally well suited for investigating the spatiotemporal distribution of MRSA clones [8]. Previous time-scaled phylogenies of historic pandemic clone ST239 have illuminated its global dissemination routes (e.g., “Turkish clade” spread into Asia) and estimated the emergence of key MRSA lineages decades in the past [9,10]. Researchers now routinely sequence MRSA genomes and use high-resolution SNP phylogenies to investigate transmission clusters in hospitals and communities, an approach that can discern recent transmission links missed by MLST or PFGE [11,12].

Although previous studies have monitored MRSA prevalent clones using MLST, they were limited in reconstructing the long-term evolutionary history and precise transmission dynamics of Korean-specific lineages due to the low resolution of traditional typing methods. Furthermore, most genomic studies in Asia have focused on short-term outbreaks or specific STs, lacking a comprehensive time-scaled evolutionary framework that covers decades of endemic circulation.

In this study, high-resolution evolutionary reconstruction of Korean MRSA was condected by analyzing comprehensive collection of clinical isolates spanning 26 years (1999–2025). We integrated time-scaled phylogenomic analysis with core genome multilocus sequence typing (cgMLST) to elucidate the distinct divergence times and adaptive evolution of major Korean clones, including ST5 and ST72. Specifically, we demonstrate the unique evolutionary trajectory of Korean lineages distinct from global strains and define the temporal emergence of antimicrobial resistance profiles.

## 2. Results

### 2.1. SNP-Based Phylogenomic Analysis

**Figure 1:**
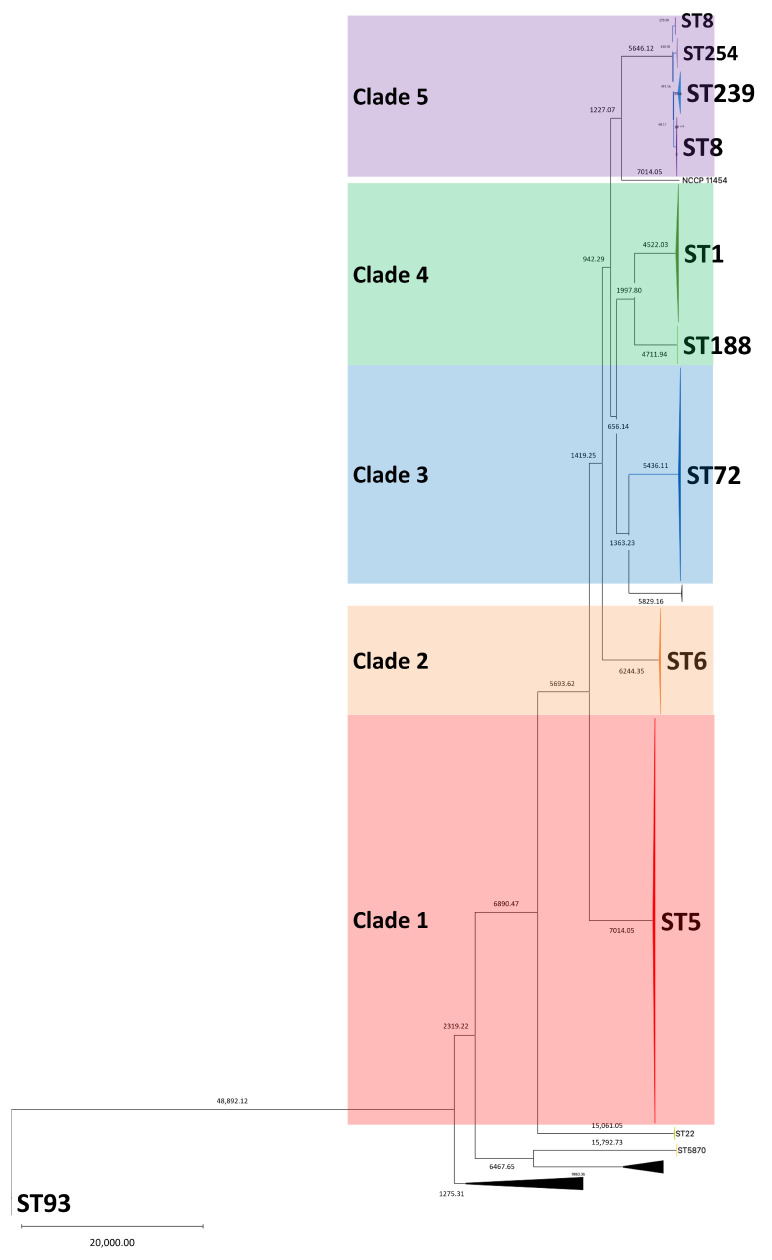
Core SNP based phylogenetic tree of MRSA isolates collected in Republic of Korea during 1999 and 2025. A total of five major clades are identified.

To investigate the distribution and genetic relatedness of the MRSA isolates collected in Republic of Korea, SNP-based phylogenetic reconstruction was performed. This analysis revealed that isolates belonging to the same MLST type clustered closely together. Five major clades were identified along with several minor lineages that could not be clearly assigned to any of the defined clades (Figure 1). Clade 1 primarily consisted of ST5 isolates; ST632 strains were also included. Clade 2 was composed exclusively of the ST6 isolates. Clade 3 included the isolates belonging to ST72, ST20, and ST513. Clade 4 comprised strains ST1 and ST188. Clade 5 contained ST8, ST239, ST254, and ST889. ST254 was nested within the ST8 cluster, whereas ST889 was embedded within the ST239 lineage. Outside of these major clades, a group of distantly related isolates formed the outer group. Among these, ST93 was positioned at the most distant edge of the phylogeny. Additional sequence types such as ST22, ST1232, and ST5870 were also distributed throughout this peripheral group.

### 2.2. Time-Scaled Epidemiologic Analysis

**Figure 2:**
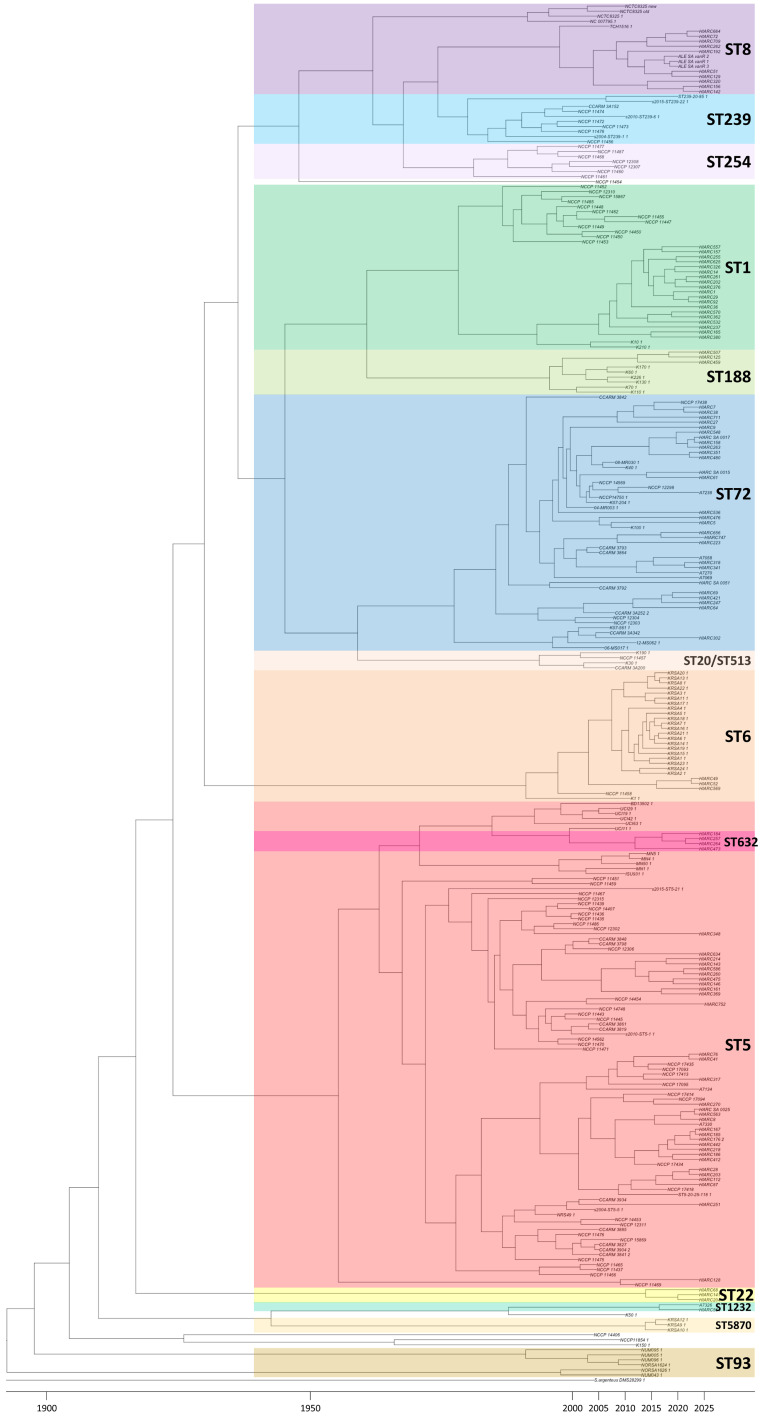
Time-scaled phylogeny of MRSA isolates. Subsequent divergence events have been estimated as follows: Clade 1 in 1918, Clade 2 in 1930, Clade 5 in 1937, and Clades 3 and 4 in 1945. Within Clade 1, Asian ST5 lineage further diverges out from the America lineage around 1963. In Clade 3, the divergence between ST72 and the ST20/ST513 lineage is estimated to have occurred around 1959. For ST1 in Clade 4, divergence between the old lineage (~2010) and the recent group (2024) occurs around 1961. In Clade 5, methicillin-susceptible *S. aureus* (MSSA) ST8 (NCTC8325) is estimated to have diverged earlier than ST239 and ST254 in 1962.

A time-scale phylogenetic analysis was conducted using the SNP-based phylogenetic tree and year-of-isolation metadata (Table 1). The results indicated that the five major clades (Clades 1–5) began to diverge from the outer group around 1916 (95% credible interval [CI]: 1812–1942). Subsequent divergence events were estimated as follows: clade 1, 1918 [95% CI: 1866–1937]; clade 2, 1930 [95% CI: 1887–1958]; clade 5, 1937 [95% CI: 1895–1953]; and clades 3 and 4, 1945 [95% CI: 1909–1961] (Figure 2). Within Clade 1, the ST5 strains further diverged into two distinct sublineages around 1963 [95% CI: 1940–1978]. The lower branch predominantly consisted of isolates from Republic of Korea, whereas the upper branch contained primarily US-origin ST5 strains, including ST632, which were clustered within this group. In Clade 3, the divergence between ST72 and the ST20/ST513 lineage was estimated to have occurred in 1959 [95% CI: 1917–1966]. For Clade 4, the divergence between ST1 and ST188 occurred around 1961 [95% CI: 1930–1973], followed by a subsequent split into two major subgroups in 1978 [95% CI: 1943–2011]. The subgroup located in the upper portion of the tree primarily included isolates collected before 2010s, while the subgroup in the lower portion mainly comprised isolates collected from 2020 and beyond. In Clade 5, methicillin-susceptible *S. aureus* ST8 (NCTC8325) was estimated to have diverged earlier than ST239 and ST254 in 1962 [95% CI: 1924–1968]. Subsequently, ST254 and ST239 emerged in 1968 [95% confidence interval [CI], 1951–1982] and 1974 [95% CI, 1936–1977], respectively.

### 2.3. Population Structure Analysis

**Figure 3:**
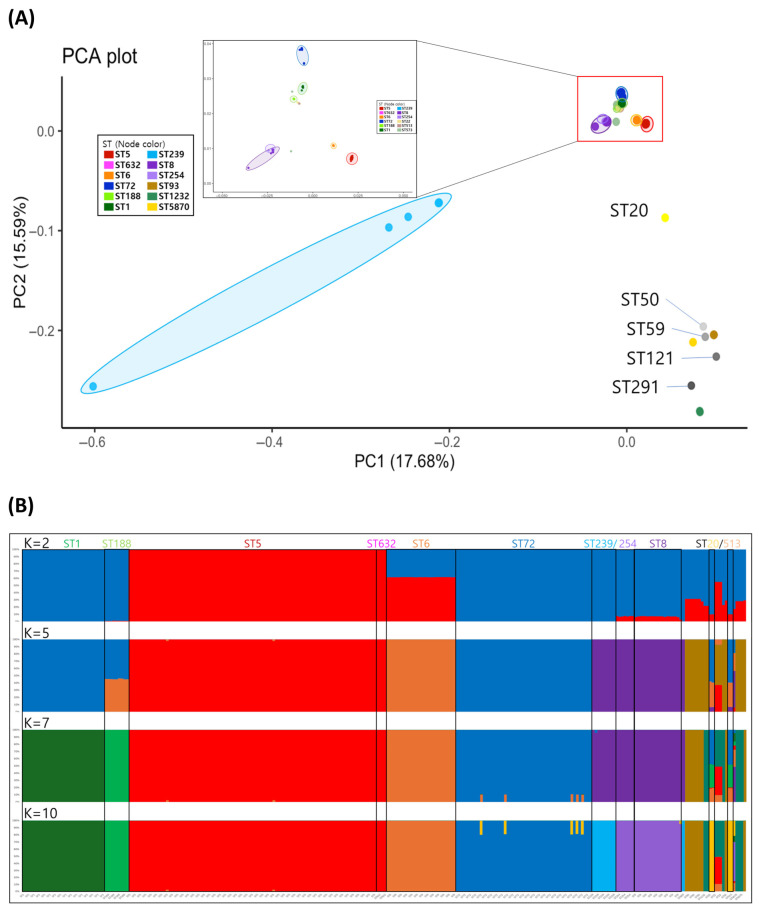
(**A**) PCA plots for population stratification. Isolates from Clades 1 through 5 are clustered closely together, whereas those from the outer clade are clearly separated and distantly positioned in the PCA space Notably, ST239 exhibits a dispersed distribution pattern, spread broadly in one direction. (**B**) ADMIXTURE-based ancestral inference for each isolate obtained via different predicted population number (K). ADMIXTURE-based ancestral inference supports time-scaled phylogeny. At K = 2, Clade 1 is inferred to share a distinct common ancestor from Clades 3, 4, and 5. At K = 5, Clades 2 and 5 emerge as independent lineages; at K = 7, Clades 3 and 4 are associated with separate ancestral components.

The gPCA results were largely consistent with SNP-based phylogenetic reconstruction. Isolates from Clades 1 to 5 clustered closely, whereas those from the outer group were clearly separated and distantly positioned in PCA space (Figure 3A). Notably, ST239 exhibited a dispersed distribution pattern, was spread broadly in one direction, and did not form cohesive clusters within any group, suggesting considerable genetic heterogeneity. Within the main clusters corresponding to clades 1–5, subclustering by sequence type was evident. Among these, Clade 1 was positioned near clade 2, whereas clade 3 was relatively close to clade 4. The ADMIXTURE analysis further supported these temporal and phylogenetic findings. At K = 2, Clade 1 was distinguished from Clades 3, 4, and 5, indicating early ancestral divergence. At K = 5, clades 2 and 5 were inferred to have distinct ancestral origins. At K = 7, clades 3 and 4 emerged as genetically distinct lineages (Figure 3B). Finally, at K = 10, a fine-scale substructure became apparent; within clade 3, ST72 diverged from ST20 and ST513; in Clade 4, ST1 and ST188 were separated; and in clade 5, ST8 and ST254 formed a group distinct from ST239. At this level, the cross-validation (CV) error is the lowest (Figure S1), supporting the selection of K = 10 as the most appropriate model.

### 2.4. Allelic Distance Analysis Based on cgMLST

**Figure 4:**
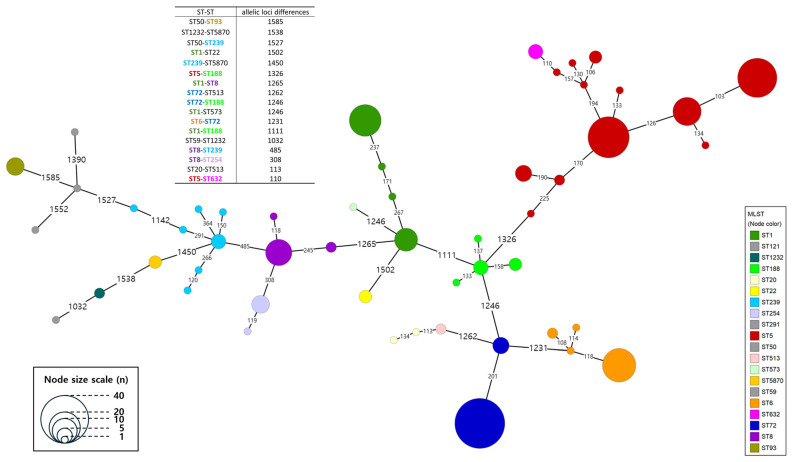
Minimum spanning tree (MST) based on cgMLST typing. Digits above the branches represent the number of allelic loci differences, and branches < 100 have been collapsed. Isolates belonging to different STs generally exhibit over 1000 allelic differences across the 1716 loci; meanwhile, notable exceptions are observed within Clade 5, where ST8 shows fewer than 500 allelic differences compared to both ST239 and ST254. Within ST5, three big sub-lineages (C.1.1, C.1.2, and C.1.3) and other small sub-lineages are observed, while ST72 exhibits only two sub-lineages (C.3.1 and C.3.2).

MST analysis based on cgMLST revealed that isolates belonging to different STs generally exhibited over 1000 allelic differences across 1716 loci (Figure 4). However, notable exceptions were observed within Clade 5, where ST8 showed fewer than 500 allelic differences from both ST239 and ST254 and between ST5 and ST632, where fewer than 200 allelic differences were identified. Within ST5, at least 12 distinct sublineages were defined using a threshold of >100 allelic differences. Among them, the three largest sublineages, designated as C.1.1, C.1.2, and C.1.3, were derived from the lower branch of the time-scaled phylogeny, which predominantly consisted of domestic isolates. For ST72, only two sublineages, C.3.1, and C.3.2, exceeded the >100 allelic difference threshold, indicating a relatively limited genetic diversity within this lineage. In ST1, the two subgroups divided by time-scaled phylogeny were also distinguishable in the MST as distinct sub-lineages, C.4.1 (including isolates before the 2010s) and C.4.2 (including isolates after 2020). One ST239 isolate, NCCP 11472, displayed over 1000 allelic differences from other ST239 isolates.

### 2.5. Distribution of Antimicrobial Resistance and Virulence Genes

**Figure 5:**
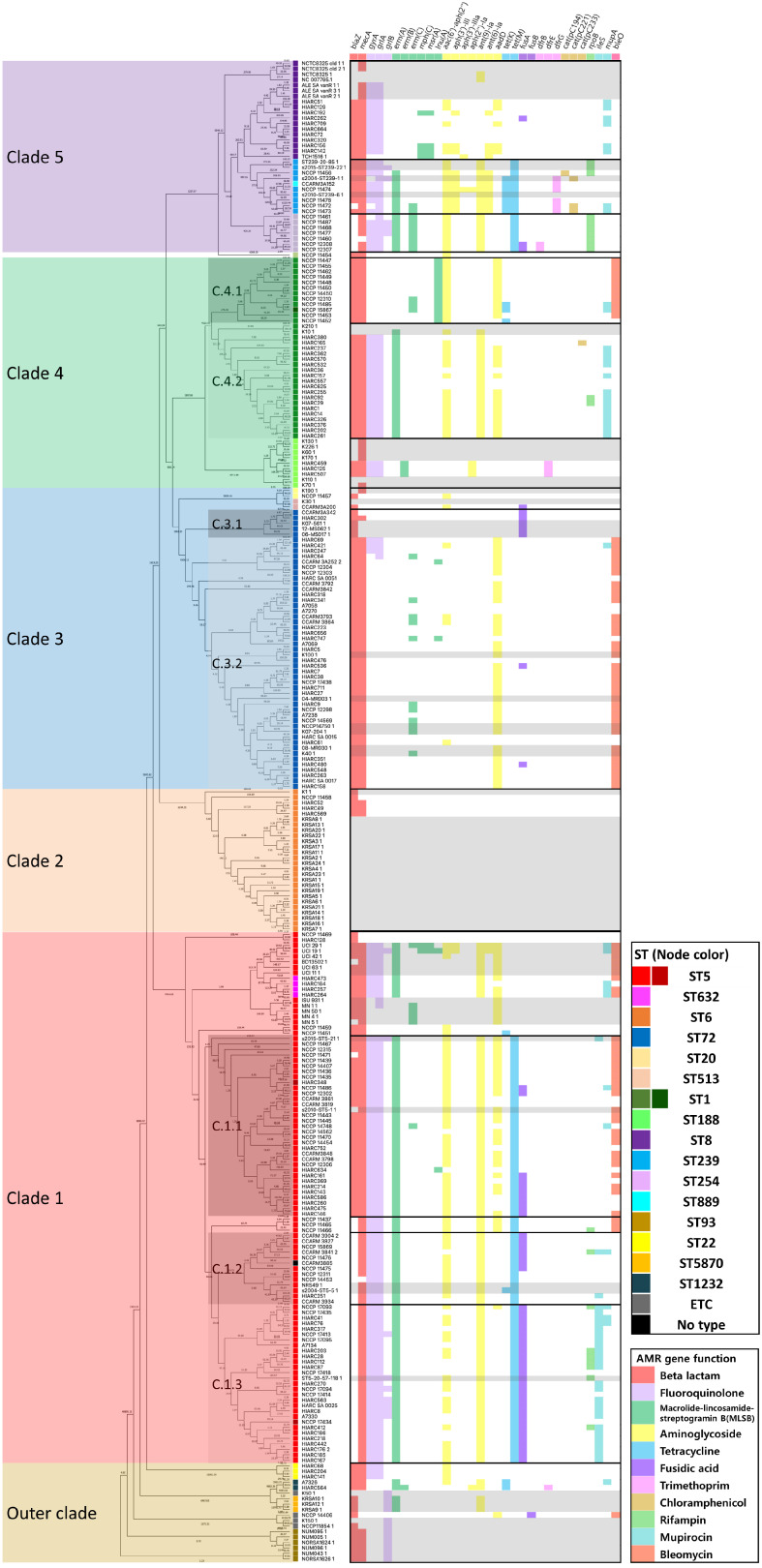
Profiling of antimicrobial resistance (AMR) factor genes. Individuals with gray shade refer to genomes collected from the sequence read archive (SRA) database of National Center for Bioinformatics (NCBI).

The virulence genes did not show a clear lineage- or ST-specific pattern across the isolates (Figure S2). In contrast, AMR genes exhibited distinct profiles among different STs, particularly at the clade level (Figure 5). Overall, Clades 1 and 5 harbored a broader spectrum of AMR genes, whereas clades 2 and 3 harbored relatively limited numbers of resistance determinants. In Clade 1, distinct AMR profiles were observed between the lower sub-lineage (mainly composed of domestic ST5 isolates) and the upper sub-lineage (including ST632 and overseas ST5 isolates). The lower group frequently harbored *blaZ*, *mecA*, *gyrA*, *grlA*, *erm(A)*, *aac(6′)-aph(2″)*, *ant(9)-Ia*, and *tet(M)*. Among the three major sublineages defined by cgMLST (C.1.1, C.1.2, and C.1.3), slight differences were observed. C.1.1 uniquely possessed *blaZ*, *aadD*, and *bleO*, whereas C.1.3 exclusively harbored *ileS*. The AMR profile of ST632 was similar to those of several ST5 reference strains (BD13502, UCI11, UCI19, UCI29, UCI42, and UCI63). Within Clade 3, significant differences in AMR gene composition were observed between the two ST72 sub-lineages, C.3.1 and C.3.2. Most of the C.3.2 isolates carried *blaZ*, *mecA*, *aadD*, and *bleO*, while C.3.1 isolates carried only two genes, *blaZ* and *fusA*. ST20 and ST513 showed fewer than three AMR genes; however, one ST513 strain (CCARM3A200) shared similar profiles with ST72 sub-lineage C.3.1. In Clade 4, ST1 and ST188 exhibited distinct resistance profiles. Comparing the older lineage (C.4.1) and the more recent lineage (C.4.2) of ST1, both shared *blaZ*, *mecA*, *aac(6′)-aph(2″)*, and *aadD*. However, C.4.1 showed lower frequencies of fluoroquinolone resistance genes (*gyrA* and grlA) and *ant(9)-Ia* and a preferential presence of *lnu(A)* over erm(A). In contrast, C.4.2 lacked *bleO*, which was more common than C.4.1. In Clade 5, ST8 harbored fewer AMR genes than ST239 and ST254, but shared *blaZ*, *mecA*, *gyrA*, and *grlA*. ST239 carried the highest diversity of resistance genes, with most isolates sharing *blaZ*, *mecA*, *grlA*, *erm(A)*, *aac(6′)-aph(2″)*, *aph(3′)-III*, *ant(9)-Ia*, *ant(6)-Ia*, *tet(K)*, *tet(M)*, and *dfrG*. ST254 also possessed a diverse AMR gene set, distinct from both ST8 and ST239, including *blaZ*, *mecA*, *grlA*, *erm(A)*, *erm(C)*, *aac(6′)-aph(2″)*, *ant(9)-Ia*, *tet(M)*, and *rpoB*. Finally, most reference isolates from ST6 (clade 2) and ST188 (clade 4) lacked detectable AMR genes in the present dataset, limiting meaningful comparisons of these lineages.

## 3. Discussion

SNP-based phylogenetic analysis revealed that the MRSA isolates collected in Republic of Korea were broadly categorized into five major clades, with most isolates clustering tightly according to their STs, demonstrating clear phylogenetic separation (Figure 1). This clustering pattern was consistent with the findings of previous studies on MRSA population structures in Asia and globally [9]. Notable exceptions included ST632, which clustered within the ST5 lineage and ST889, which clustered within the ST239 group. These exceptions have also been reported in earlier studies [13]. Beyond the major clades, a small number of isolates belonging to ST that are less prevalent in Republic of Korea, such as ST22 and ST1232, were also identified. However, due to the limited number of isolates, no statistically meaningful phylogenetic or epidemiological trends could be determined. Notably, ST72 strains did not cluster within clade 5, which contained canonical CC8 members ST8, ST239, and ST254. Instead, ST72 formed an independent cluster (clade 3) and was phylogenetically closer to clade 4, which comprised ST1 and ST188 (CC1). This pattern of divergence supports previous studies that reported genotypic similarities and distinct evolutionary paths between ST72 and other CC8 lineages [14,15].

Time-scaled phylogenetic reconstruction indicated that clade 1 diverged first around 1918, followed by clade 2 in 1930, clade 5 in 1937, and clades 3 and 4 in 1945 (Figure 2). This temporal progression was further supported by the ADMIXTURE-based ancestral inference. At K = 2, clade 1 was inferred to share a distinct common ancestor with clades 3, 4, and 5. At K = 5, clades 2 and 5 emerged as independent lineages, and at K = 7, clades 3 and 4 were associated with separate ancestral components (Figure 3B), mirroring the evolutionary split inferred from the timescale tree. When examined at the individual sequence type (ST) level, distinct evolutionary signatures were observed within each major clade. These lineage-specific characteristics warrant a more detailed investigation into their divergence history and resistance gene dynamics.

Consistent with our aim to define the unique evolutionary trajectory of Korean lineages, the most prevalent ST, ST5, showed a distinct separation from global strains. The MRSA of ST5 is estimated to have emerged in the 1940s. This lineage is known to have caused widespread dissemination in Asia, including Republic of Korea, during the 1990s, whereas in the United States, it likely circulated earlier, from the 1980s to the early 1990s [16]. In our study, ST5 isolates originating from Republic of Korea and other Asian countries formed a distinct phylogenetic cluster that was clearly separated from the North American group, which included isolates from Iowa State University (ISU), University of Minnesota (MN), and University of California, Irvine (UCI), as shown in both the SNP-based phylogeny and cgMLST analyses (Figure 4) [17]. Within the Korean/Asian ST5 cluster, three sub-lineages, C.1.1, C.1.2, and C.1.3, were defined based on cgMLST allelic distances and AMR gene profiles (Figure 5).

A previous global comparative study identified five major lineages (hereafter referred to as ‘prior-clades 1–5’ to distinguish them from this study’s Clades). In that classification, North American MRSA isolates were grouped into prior-clade 1, whereas Asian strains fell into prior-clades 2 and 3. In that study, MRSA isolates from Asia and North America were predominantly found in prior-clades 4 and 5, respectively [16]. By comparing the AMR gene profiles, the sub-lineages identified in our study were inferred to correspond to those previously described. The North American cluster, characterized by the presence of *blaZ* and *aadD* and the general absence of *tet(M)*, matched the features of prior-clade 1 in a previous study. Furthermore, this group showed distinct virulence gene patterns, such as the presence or absence of *scn* and *tst*, which aligned with the characteristics of prior-clade 1’s characteristics.

A notable distinction between prior-clade 1 and prior-clades 2/3, which represent the North American and Asian ST5 MRSA lineages, was the presence or absence of *tet(M)* and *tet(K)* genes. The *tet(M)* encodes a ribosomal protective protein that displaces tetracycline from the bacterial ribosome, whereas *tet(K)* encodes an efflux pump that actively transports tetracycline out of the cell [18]. Although not assessed in the current study, previous studies have identified other tetracycline resistance genes such as *tet(T)* and *tet(L)*, which function similarly to *tet(M)* and *tet(K)*, respectively. Notably, *tet(T)* and *tet(L)* were detected in isolates from Iowa State University (ISU) and the University of Minnesota (MN) but were absent in strains from the University of California, Irvine (UCI) [17]. The estimated divergence time of Asian and North American ST5 sublineages (around 1963) predates the widespread clinical introduction of tetracycline in 1978 [19]. This temporal incongruence provides compelling evidence that tetracycline resistance was not inherited from a common ancestor but was acquired independently through horizontal gene transfer (HGT) post-divergence.

Specifically, the predominance of *tet(M)* (ribosomal protection) in Korean isolates versus *tet(K)* or *tet(L)* (efflux pumps) in North American strains reflects distinct adaptive strategies driven by the local availability of mobile genetic elements and varying antibiotic prescribing practices in each region. The acquisition of *tet(M)* in the Asian lineage likely occurred because this determinant was circulating within the specific plasmid or transposon pool of Asian healthcare settings during the expansion of the ST5 clone. This reinforces the hypothesis that while the core genome evolved globally, the resistome was shaped by region-specific antibiotic selection pressures.

Within the Korean/Asian ST5 cluster, sublineage C.1.1 was characterized by the presence of *blaZ* and *aadD* with only partial acquisition of *tet(M)*, making it a suitable match for the previously defined clade 3. In contrast, C.1.2 and C.1.3 lacked *blaZ* and *aadD* but consistently harbored *tet(M)*, suggesting a correspondence with clade 2. Because methicillin-susceptible *S. aureus* (MSSA) strains were not included in this study, lineages corresponding to clades 4 and 5, which were defined in part by MSSA profiles in earlier studies, could not be inferred.

Previous studies on ST5-MRSA, particularly clade II, have reported structural deletions within the SCC*mec* type II element, specifically the loss of *ble* and *knt*, resulting in shorter cassette size [16]. This streamlining may have contributed to increased fitness and facilitated clonal expansion, especially within Chinese MRSA populations. Studies have suggested that SCC*mec* elements smaller than 45 kb may be transferred by bacteriophage transduction, whereas larger elements have a limited transfer mechanism [20,21,22]. In line with this hypothesis, our findings revealed that sub-lineages C.1.2 and C.1.3 lacked the *bleO* gene, which was present in C.1.1. Notably, the isolates within C.1.3 were mostly recovered after 2015, suggesting that these are relatively recent derivatives that may have undergone similar cassette simplifications. This evidence supports the idea that the C.1.2/C.1.3 lineage emerged through structural loss events, including deletions within the SCC*mec* element and the loss of plasmid-encoded resistance genes. Following this streamlining, selective pressure from the clinical use of antibiotics such as mupirocin and rifampin may have driven the acquisition of resistance genes (such as *ileS* and *rpoB)* in these sublineages [23,24].

Additionally, ST632 clustered closely with overseas ST5 strains, particularly those from UCI, in both phylogenetic and AMR profile analyses. This close relationship strongly suggests that ST632 represents a recently diverged foreign clone introduced into Republic of Korea [17].

Although the overall sub-lineage structure and resistance patterns were consistent with previous findings, there were slight differences in the estimated divergence dates. A prior study suggested that the North American ST5 clade diverged around 1945, with clades 2 and 3 splitting around 1965 [16]. In contrast, our temporal analysis estimated the divergence of the North American cluster at approximately 1963 and the subsequent split of C.1.1 from C.1.2/C.1.3 around 1972 (Figure 2). These temporal differences reflect a methodological distinction in dataset composition. While earlier studies included diverse MSSA strains to reconstruct deep ancestry, our study focused exclusively on clinical MRSA isolates. Therefore, our divergence estimates likely reflect the timing of ‘resistance expansion’ and local transmission events within the healthcare system, rather than the initial emergence of the lineage from a susceptible ancestor. This exclusion of the deep MSSA reservoir naturally results in more recent (delayed) estimates for the most recent common ancestor (MRCA) of the resistant clades.

ST72 is prevalent in Republic of Korea. It has become the increasingly dominant community-associated MRSA (CA-MRSA) lineage in Republic of Korea since its emergence in the early 2000s [6,7]. Regarding the endemic stability of Korean clones, ST72 isolates were previously categorized into four distinct groups (hereafter ‘prior-clades A, B, C, and D’), based on core genome phylogeny and accessory gene content [20]. Notably, prior-clade D consisted exclusively of MSSA, whereas Korean MRSA isolates were reported to be confined to prior-clades A and C. In our analysis, the Korean ST72 MRSA isolates similarly segregated into two subgroups, C.3.1 (including isolate K07-561) and C.3.2 (including isolate K07-204), based on cgMLST and AMR gene profiles. This sublineage structure is consistent with that of previous studies and reinforces the notion that only a subset of ST72 diversity contributes to MRSA dissemination in Republic of Korea.

Importantly, prior-clade A isolates were previously reported to harbor fewer AMR genes than prior-clade B isolates, but only carried the *fusA* gene. In our dataset, the C.3.1 cluster (corresponding to prior-clade A) similarly exhibited a lower AMR gene burden than C.3.2, yet was the only subgroup to possess *fusA* (Figure 5). These findings suggest conserved evolutionary patterns within ST72 MRSA sublineages circulating in Republic of Korea and highlight the utility of resistance gene content as a marker for sublineage differentiation.

A previous phylogenomic study suggested that ST72 prior-lade A diverged as early as 1939 (95% HPD: 1906–1960), preceding the widespread use of antibiotics, whereas prior-clade C was inferred to have separated from the MSSA-dominated prior-clade D at a later point. This led us to hypothesize that prior-clades A and C acquired antimicrobial resistance independently through distinct evolutionary trajectories [20]. In our analysis, cgMLST clearly differentiated the two Korean MRSA subgroups, C.3.1 and C.3.2 (Figure 4), yet molecular dating inferred that their divergence occurred in the mid-1950s (Figure 2), somewhat later than the estimates reported in earlier studies. As with the divergence estimates for ST5 sublineages, these differences are likely attributable to variations in dataset composition, particularly the exclusion of MSSA isolates and the inclusion of multiple STs in a single phylogenetic framework.

Notably, ST72 (especially the C.3.1 cluster), ST20, and ST513 isolates were in close genetic proximity. Genotypic similarities between ST72 and ST513 have been noted, suggesting a possible shared evolutionary background or convergent adaptation [14,15]. In our dataset, strain CCARM3A200 (ST513) exhibited AMR gene profiles nearly identical to those of C.3.1 ST72 strains, even though this isolate was distantly positioned in other phylogenetic analyses, indicating a lack of recent common ancestry. In contrast, the other ST20 and ST513 isolates were closely positioned in phylogenetic analysis, but none shared similar AMR profiles. This genomic inconsistency suggests that horizontal gene transfer (HGT), rather than vertical inheritance, may explain the similarity in AMR gene content between these lineages. Such putative HGT events between ST72 and ST513 could reflect shared environmental or host-associated selective pressures, such as antibiotic exposure or colonization niches, which drive the exchange of mobile genetic elements that confer resistance.

Although ST1 is not a major type in Republic of Korea, plenty of isolates were collected in this study. According to previous epidemiological studies, the ST1 MRSA lineage first emerged in Europe around 1995 and subsequently spread throughout the late 1990s and the 2000s as CC1-MRSA-IV [25], The overall genomic diversity of this lineage has been reported to be relatively limited. In the United States, ST1 is associated with the USA400 clone, which circulated in the 1990s prior to the emergence of USA300. In our molecular dating analysis, the ST1 lineage appeared to have undergone bifurcation around 1978, resulting in two distinct sublineages: C.4.1 (earlier) and C.4.2 (more recent) (Figure 2). The expansion of C.4.2 likely coincides with the acquisition of a broader array of antimicrobial resistance (AMR) genes, suggesting adaptive diversification under antibiotic selection pressure. Compared to C.4.1, C.4.2, isolates exhibited resistance to a wider range of antibiotic classes, particularly fluoroquinolones, which was not observed in the earlier lineage (Figure 5).

In terms of macrolide resistance, C.4.2 strains carried *erm(A)* instead of *lnu(A)*, which is typically found in C.4.1. The *erm(A)* gene encodes a methyltransferase that modifies 23S rRNA and confers broad resistance to macrolide-lincosamide-streptogramin B (MLSB) antibiotics. In contrast, *lnu(A)* encodes a lincosamide nucleotidyltransferase that adenylates and inactivates only a narrow range of lincosamides, thus offering less comprehensive protection [26,27]. Additionally, C.4.2 isolates harbored other resistance determinants, including *ant(9)-Ia* (aminoglycoside resistance) and *mup(A)* (mupirocin resistance) [23,24], which were absent in C.4.1. These findings support the hypothesis that C.4.2 represents a more recent sublineage that has acquired resistance through local antibiotic exposure in Republic of Korea. Notably, not all isolates within C.4.2 exhibited a high resistance gene content. For example, isolates K10 and K210 carried few or no AMR genes. This suggests that, while the C.4.2 cluster is defined by enhanced resistance, resistance acquisition may have occurred gradually and heterogeneously within this lineage in response to variable antimicrobial pressures. Given these patterns and distinct phylogenetic and resistance profiles, C.4.2 appears to have diversified independently of the European CC1-MRSA-IV lineage.

ST8 MRSA was introduced into North America in the early 20th century and subsequently acquired SCC*mec* type IV and the arginine catabolic mobile element (ACME) in the latter half of the century [28], The pre-epidemic evolutionary trajectory of this lineage, particularly the emergence of the highly transmissible USA300 clone, has been thoroughly described in a recent phylogenomic study [29]. According to this study, the North American epidemic (NAE) and South American epidemic (SAE) clades diverged from a common ancestor around 1970 (95% HPD: 1966–1974), indicating that a transmissibility-primed progenitor lineage existed in the late 1960s.

Our timescale phylogenetic reconstruction supported this timeline. Within Clade 5, MSSA ST8 was inferred to have diverged first around 1962, followed by the emergence of ST254 in 1968 and ST239 in 1974 (Figure 2). This ordering is consistent with the evolutionary backdrop proposed for USA300 and the related epidemic lineages. Although the ST8 isolates in our dataset harbored fewer AMR genes than ST254 and ST239, they consistently shared several key resistance determinants, including *blaZ*, *mecA*, *gyrA*, and *grlA* (Figure 5). These shared features suggest that, despite the lower resistance gene burden, ST8 retains the essential determinants of methicillin and fluoroquinolone resistance, which may support its persistence and epidemiological success in various contexts.

Historically, ST239 MRSA was responsible for major hospital outbreaks in Taiwan during 1989, 1993, and 1995 and dominated nosocomial MRSA infections in Republic of Korea throughout the 1990s and the 2000s. A recent hypothesis proposed that ST239 originated from a large-scale chromosomal recombination event between ST8 and ST30 that occurred between 1920 and 1945 [10]. According to this study, approximately 20% (~600 kb) of the ST239 genome is derived from ST30, whereas the remainder (including the core genome) is closely related to ST8. Supporting this, pairwise SNP distance analyses showed a high similarity between ST239 and ST30 when focusing on specific genomic regions, whereas core genome-based comparisons placed ST239 much closer to ST8. In our own inspection using Integrative Genomics Viewer (IGV), we identified two SNP-dense regions in ST239 isolates: 0–320 kb and 2.64–2.82 Mb, collectively spanning approximately 500 kb, which corresponds well with the reported ST30-derived segments (Figure S3).

This origin of recombination explains the anomalous results observed in the phylogenetic analysis. For example, PCA, which does not specifically adjust for recombination or localized hypervariability, placed ST239 unusually far from the ST8 cluster (Figure 3A). In contrast, ADMIXTURE, which evaluates the average ancestral contribution across the entire genome, is more robust against these distortions. At K = 7, ST239 still clustered with ST8, suggesting a shared ancestral background, despite the mosaic structure of the genome (Figure 3B). Moreover, owing to the high similarity between ST239’s core genome of ST8, the cgMLST-based distance between them was shorter than that between many other STs. However, our time-scaled phylogeny estimated the divergence of ST239 from ST8 to be around 1974, which was later than that of ST254 (Figure 2). This discrepancy may stem from insufficient correction for recombinational and hypervariable regions, which can compress the apparent divergence times. Given the extensive recombination-derived structure of ST239, its true evolutionary origin is likely traced back to the 1920–1945 window as previously proposed.

Although the majority of ST239 isolates formed a cohesive cluster across SNP-based phylogeny, cgMLST, and AMR profiles, some intra-lineage heterogeneity was observed. NCCP11472 appeared to be an outlier in both the PCA- and cgMLST-based analyses, exhibiting substantial divergence from other ST239 strains. This strain likely harbored an elevated proportion of recombined genomic content derived from ST30, potentially extending into the loci used in the cgMLST scheme, thereby exaggerating its distance from other ST239 isolates. Conversely, although ST889 (CCARM3A152) was assigned a different ST by MLST, it displayed near-complete concordance with ST239 in SNP phylogeny, cgMLST profiles, and AMR gene composition. This finding supports the interpretation that ST889 falls within the natural diversity of the ST239 lineage and may represent a sub-lineage variant rather than a distinct evolutionary branch.

Despite its historically high resistance burden, the prevalence of ST239 has declined dramatically in Republic of Korea since 2010, as reported by previous surveillance studies. This paradox of high resistance yet declining dominance may be explained by the reduced overall fitness or adaptability of ST239 relative to other clones, such as ST8 or ST30, a phenomenon also noted in global epidemiological analyses. In support of this, only a small proportion of our recently collected clinical isolates belonged to ST239, further confirming its waning presence in the contemporary Korean MRSA landscape.

This study has several limitations. First, regarding dataset composition, this study was designed to focus specifically on the evolutionary history of resistant clones (MRSA) circulating in the Korean healthcare system. Consequently, the exclusion of the diverse MSSA reservoir implies that our divergence time estimates reflect the timeline of MRSA lineage expansion and resistance consolidation rather than the deep ancestry of the *S. aureus* species. Therefore, the observation of more recent (delayed) divergence dates compared to studies including MSSA backgrounds is an expected outcome of this targeted sampling scope. Second, sampling bias in recent years (e.g., 2024) and a relative lack of isolates from 2010 to 2020 may have influenced the precision of molecular clock estimations. Finally, although phenotypic antimicrobial susceptibility testing (AST) has been conducted for these isolates, these data were not included in the current analysis to maintain the focus on phylodynamic reconstruction. While WGS-based resistance prediction is generally highly concordant with phenotype, detailed genotype-phenotype correlation analyses are required to confirm expression levels. These phenotypic validations and discordance analyses are currently the subject of a follow-up study.

## 4. Materials and Methods

### 4.1. Isolates Collection

A total of 191 clinical MRSA isolates collected between 1999 and 2025 were randomly obtained from four major Korean biobanks: the Antibiotic Resistance Specialized Pathogen Resource Bank (n = 101), National Culture Collection for Pathogens (n = 66), Culture Collection of Antimicrobial-Resistant Microbes (n = 17), and Asan Medical Center (n = 7). These repositories aggregate clinical isolates from secondary and tertiary hospitals located across various provinces in Republic of Korea. Therefore, the dataset provides a nationwide geographic representation and minimizes the selection bias typically associated with single-center studies. Details of the isolates are provided in Table 1. All isolates were stored at −80 °C until further analysis.

### 4.2. Genomic DNA Extraction and Whole-Genome Library Preparation

Genomic DNA (gDNA) was extracted using a Quick-DNA Fungal/Bacterial Miniprep Kit (Zymo Research, Tustin, CA, USA) following the manufacturer’s protocol. The concentration and quality of the extracted DNA were evaluated by NanoDrop spectrophotometry (Wilmington, DE, USA), Qubit fluorometry, and 1% agarose gel electrophoresis. High-molecular-weight DNA was subsequently fragmented using a Qsonica Q800R sonicator (Qsonica, Newtown, CT, USA), and the resulting DNA fragments were purified using a QIAquick PCR Purification Kit (Qiagen, Hilden, Germany). Sequencing libraries were constructed using the VAHTS Universal DNA Library Prep Kit for Illumina v4 (Vazyme, Nanjing, China), which included enzymatic end repair, adapter ligation, and size selection for fragments of approximately 300–500 bp. Paired-end sequencing (2 × 150 bp) was performed using an Illumina NextSeq 1000 platform (Illumina, San Diego, CA, USA).

### 4.3. Read Processing and Assembly

For comparative genomic analysis, 77 genomes representing diverse STs, including ST1, ST5, ST6, ST8, ST20, ST50, ST72, ST93, ST188, ST239, ST291, ST513, and ST5870, were retrieved from the NCBI Sequence Read Archive (SRA) as Illumina short-read datasets (Table 2). All reference datasets were subjected to the same analytical pipeline used for the isolates in this study. Raw reads were quality- and adapter-trimmed and subsequently assembled de novo using the CLC Genomics Workbench v22.0 (QIAGEN, Germany) workflow (Figure S4) [30,31,32,33]. Recombination regions were detected and masked using GUBBINS v3.2.1 (Genealogies Unbiased By recomBinations In Nucleotide Sequences) [34]. Phylogenetic relationships were inferred using RAxML v8 [35], and the resulting tree was visualized using MEGA v12. In addition, reference-based single nucleotide polymorphism (SNP) mapping was performed using the *S. aureus* reference genome, NCTC8325 (NC_007795.1).

### 4.4. Time-Scaled Epidemiologic Analysis

To perform a time-scaled phylogenetic analysis, sampling years and an SNP-based phylogenetic tree (Newick format) were used as input data for BactDating v1.1 [36]. Markov chain Monte Carlo (MCMC) simulations were conducted under the ‘arc’ model, specifically designed for bacterial evolution, to perform Bayesian time-scaled analysis. Posterior estimates derived from the Markov chain MCMC output were used to calculate the evolutionary rate, estimate the time to the most recent common ancestor (MRCA), and determine lineage divergence times.

### 4.5. Genome-Wide Association Study (GWAS) for Population-Structure Analyses

Variant calling results obtained from reference-based SNP mapping were exported in Variant Call Format (VCF). A GWAS was initially conducted using PLINK (v 0.99n) for primary association scans [37]. To account for population stratification and reduce dimensionality, genomic principal component analysis (gPCA) was performed using the GCTA v1.94.1 software package [38,39]. The population structure was further investigated using ADMIXTURE v1.3.0 to estimate individual ancestry proportions and to infer the number of ancestral components [40,41]. All graphical outputs were visualized using the ggplot2 package in R v4.3.1.

### 4.6. Core Genome Multilocus Sequence Typing (cgMLST) and Minimum-Spanning Analysis

The cgMLST scheme was obtained from the PubMLST database [42]. The cgMLST typing was performed using de novo assembled genome sequences based on this standardized scheme. Allelic profiles were compiled and used to construct a minimum-spanning tree (MST) based on locus-by-locus allelic differences. A threshold of 100 allelic differences was empirically applied to define sub-lineages. This cutoff was selected based on the distribution of pairwise allelic distances observed in our dataset, where epidemiologically related clusters typically exhibited distinct separation from other lineages beyond this range, allowing for a robust definition of major evolutionary sub-groups (e.g., C.1.1, C.1.2) without over-fragmentation. MST were generated using the CLC Genomics Workbench (QIAGEN, Germany) to visualize the genetic relatedness among the isolates.

### 4.7. AMR, Virulence Finding

Functional genes associated with antimicrobial resistance and virulence were annotated using the de novo assembled genome sequences. Antimicrobial resistance genes were identified using the web-based tool ResFinder v4.7.2 (https://genepi.food.dtu.dk/resfinder, accessed on 1 February 2026) [43,44], while virulence-associated genes were detected using VirulenceFinder v2.0 (https://cge.food.dtu.dk/services/VirulenceFinder/, accessed on 1 February 2026) [45,46,47,48,49].

## 5. Conclusions

In this study, we reconstructed the long-term epidemiologic history of Korean MRSA by integrating high-resolution time-scaled phylogenomics with cgMLST. Unlike previous surveillance limited to MLST, this approach allowed us to elucidate the precise divergence times and transmission dynamics of major endemic clones. Specifically, we demonstrated that Korean lineages (ST5, ST72) followed evolutionary trajectories distinct from global strains, and we identified the temporal emergence of multidrug-resistant ST1 sub-lineages.

Our time-scaled phylogenetic analyses produced divergent estimates that were generally delayed compared to those reported in previous studies. This temporal shift is likely attributable to several factors, including the exclusion of MSSA isolates and the use of a broad multi-ST sampling framework. Additionally, sampling bias in recent years (e.g., 2024) and a lack of isolates from 2010 to 2020 may have further contributed to these discrepancies in the molecular clock estimation.

Therefore, we believe that the evolutionary and transmission scenarios inferred in this study are robust and biologically plausible, particularly when considered in parallel with prior functional and molecular evidence. Nevertheless, expanding the temporal and geographic diversity of isolates, as well as performing experimental validation (e.g., antimicrobial susceptibility testing and transcriptomic analysis), will be crucial for refining our understanding of lineage-specific adaptations and selective pressures in *S. aureus*.

## Figures and Tables

**Table 1:** List of the 191 clinical MRSA isolates collected in Republic of Korea used in this study. Annual isolate counts: ‘99–‘04 (17); ‘05–‘09 (24); ‘10–‘14 (0); ‘15–‘19 (7); ‘20–‘24 (106); ‘25 (2); ND (35). (Total N = 191).

Name	ST ^1^	Year ^2^	Origin ^3^	Name	ST	Year	Origin
CCARM_3885	ST5(NA)	2005	CCARM	NCCP_11443	ST5	2001	NCCP
HIARC001	ST1	2024	HIARC	NCCP_11445	ST5	ND	NCCP
HIARC014	ST1	2024	HIARC	NCCP_11451	ST5	ND	NCCP
HIARC029	ST1	2024	HIARC	NCCP_11459	ST5	ND	NCCP
HIARC036	ST1	2024	HIARC	NCCP_11465	ST5	ND	NCCP
HIARC092	ST1	2024	HIARC	NCCP_11466	ST5	ND	NCCP
HIARC157	ST1	2024	HIARC	NCCP_11467	ST5	ND	NCCP
HIARC165	ST1	2024	HIARC	NCCP_11469	ST5	ND	NCCP
HIARC202	ST1	2024	HIARC	NCCP_11470	ST5	2001	NCCP
HIARC237	ST1	2024	HIARC	NCCP_11471	ST5	ND	NCCP
HIARC255	ST1	2024	HIARC	NCCP_11475	ST5	2001	NCCP
HIARC261	ST1	2024	HIARC	NCCP_11476	ST5	2001	NCCP
HIARC326	ST1	2024	HIARC	NCCP_11486	ST5	2000	NCCP
HIARC362	ST1	2024	HIARC	NCCP_12302	ST5	ND	NCCP
HIARC376	ST1	2024	HIARC	NCCP_12306	ST5	ND	NCCP
HIARC380	ST1	2024	HIARC	NCCP_12311	ST5	2009	NCCP
HIARC532	ST1	2024	HIARC	NCCP_12315	ST5	ND	NCCP
HIARC557	ST1	2024	HIARC	NCCP_14407	ST5	2003	NCCP
HIARC570	ST1	2024	HIARC	NCCP_14453	ST5	2008	NCCP
HIARC625	ST1	2024	HIARC	NCCP_14454	ST5	2008	NCCP
NCCP_11448	ST1	ND	NCCP	NCCP_14562	ST5	2001	NCCP
NCCP_11449	ST1	2001	NCCP	NCCP_14748	ST5	2005	NCCP
NCCP_11450	ST1	ND	NCCP	NCCP_15869	ST5	2009	NCCP
NCCP_11452	ST1	ND	NCCP	NCCP_17093	ST5	2017	NCCP
NCCP_11453	ST1	ND	NCCP	NCCP_17094	ST5	ND	NCCP
NCCP_11455	ST1	ND	NCCP	NCCP_17095	ST5	2017	NCCP
NCCP_11462	ST1	ND	NCCP	NCCP_17413	ST5	2017	NCCP
NCCP_11485	ST1	1999	NCCP	NCCP_17414	ST5	2018	NCCP
NCCP_12310	ST1	ND	NCCP	NCCP_17418	ST5	2018	NCCP
NCCP_14450	ST1	2008	NCCP	NCCP_17435	ST5	2018	NCCP
NCCP_15867	ST1(NT)	2005	NCCP	HIARC348	ST5(NT)	2024	HIARC
NCCP_14406	ST121	ND	NCCP	NCCP_17434	ST5(NT)	2016	NCCP
A7326	ST1232	2024	Asan	CCARM3A200	ST513	2008	CCARM
HIARC564	ST1232	2024	HIARC	NCCP_11454	ST573	ND	NCCP
HIARC125	ST188	2024	HIARC	HIARC049	ST6	2024	HIARC
HIARC459	ST188	2024	HIARC	HIARC052	ST6	2024	HIARC
HIARC507	ST188	2024	HIARC	HIARC569	ST6	2024	HIARC
NCCP_11457	ST20	ND	NCCP	NCCP_11458	ST6	ND	NCCP
HIARC068	ST22	2024	HIARC	HIARC184	ST632	2024	HIARC
HIARC141	ST22	2024	HIARC	HIARC257	ST632	2024	HIARC
HIARC204	ST22	2024	HIARC	HIARC264	ST632	2024	HIARC
NCCP_11456	ST239	ND	NCCP	HIARC473	ST632	2024	HIARC
NCCP_11472	ST239	2001	NCCP	A7058	ST72	2024	Asan
NCCP_11473	ST239	ND	NCCP	A7069	ST72	2024	Asan
NCCP_11474	ST239	2001	NCCP	A7238	ST72	2024	Asan
NCCP_11478	ST239	2001	NCCP	A7270	ST72	2024	Asan
NCCP_11460	ST254	ND	NCCP	CCARM_3792	ST72	2005	CCARM
NCCP_11461	ST254	ND	NCCP	CCARM_3793	ST72	2005	CCARM
NCCP_11468	ST254	2001	NCCP	CCARM_3842	ST72	2005	CCARM
NCCP_11487	ST254	ND	NCCP	CCARM_3864	ST72	2005	CCARM
NCCP_12307	ST254	ND	NCCP	CCARM_3A252	ST72	2008	CCARM
NCCP_12308	ST254	ND	NCCP	CCARM3A342	ST72	2007	CCARM
A7134	ST5	2024	Asan	HARC_SA_0015	ST72	2024	HIARC
A7330	ST5	2024	Asan	HARC_SA_0017	ST72	2024	HIARC
CCARM_3798	ST5	2005	CCARM	HARC_SA_0051	ST72	2024	HIARC
CCARM_3819	ST5	2005	CCARM	HIARC005	ST72	2024	HIARC
CCARM_3827	ST5	2005	CCARM	HIARC007	ST72	2024	HIARC
CCARM_3841	ST5	2005	CCARM	HIARC009	ST72	2024	HIARC
CCARM_3848	ST5	2005	CCARM	HIARC027	ST72	2024	HIARC
CCARM_3861	ST5	2005	CCARM	HIARC038	ST72	2024	HIARC
CCARM_3904	ST5	2005	CCARM	HIARC061	ST72	2024	HIARC
CCARM_3934	ST5	2005	CCARM	HIARC064	ST72	2024	HIARC
HARC_SA_0025	ST5	2024	HIARC	HIARC069	ST72	2024	HIARC
HIARC008	ST5	2024	HIARC	HIARC158	ST72	2024	HIARC
HIARC028	ST5	2024	HIARC	HIARC223	ST72	2024	HIARC
HIARC041	ST5	2024	HIARC	HIARC247	ST72	2024	HIARC
HIARC076	ST5	2024	HIARC	HIARC263	ST72	2024	HIARC
HIARC087	ST5	2024	HIARC	HIARC302	ST72	2024	HIARC
HIARC112	ST5	2024	HIARC	HIARC318	ST72	2024	HIARC
HIARC128	ST5	2024	HIARC	HIARC341	ST72	2024	HIARC
HIARC143	ST5	2024	HIARC	HIARC351	ST72	2024	HIARC
HIARC146	ST5	2024	HIARC	HIARC421	ST72	2024	HIARC
HIARC161	ST5	2024	HIARC	HIARC476	ST72	2024	HIARC
HIARC167	ST5	2024	HIARC	HIARC480	ST72	2024	HIARC
HIARC176_2	ST5	2024	HIARC	HIARC536	ST72	2024	HIARC
HIARC185	ST5	2024	HIARC	HIARC548	ST72	2024	HIARC
HIARC186	ST5	2024	HIARC	HIARC655	ST72	2024	HIARC
HIARC203	ST5	2024	HIARC	HIARC711	ST72	2024	HIARC
HIARC214	ST5	2024	HIARC	HIARC747	ST72	2025	HIARC
HIARC218	ST5	2024	HIARC	NCCP_12298	ST72	ND	NCCP
HIARC251	ST5	2024	HIARC	NCCP_12303	ST72	ND	NCCP
HIARC260	ST5	2024	HIARC	NCCP_12304	ST72	ND	NCCP
HIARC270	ST5	2024	HIARC	NCCP_14569	ST72	2005	NCCP
HIARC317	ST5	2024	HIARC	NCCP_17438	ST72	ND	NCCP
HIARC369	ST5	2024	HIARC	HIARC051	ST8	2024	HIARC
HIARC412	ST5	2024	HIARC	HIARC072	ST8	2024	HIARC
HIARC442	ST5	2024	HIARC	HIARC129	ST8	2024	HIARC
HIARC475	ST5	2024	HIARC	HIARC142	ST8	2024	HIARC
HIARC563	ST5	2024	HIARC	HIARC156	ST8	2024	HIARC
HIARC586	ST5	2024	HIARC	HIARC192	ST8	2024	HIARC
HIARC634	ST5	2024	HIARC	HIARC262	ST8	2024	HIARC
HIARC752	ST5	2025	HIARC	HIARC320	ST8	2024	HIARC
NCCP_11435	ST5	2001	NCCP	HIARC664	ST8	2024	HIARC
NCCP_11436	ST5	2001	NCCP	HIARC709	ST8	2024	HIARC
NCCP_11437	ST5	ND	NCCP	CCARM3A152	ST889	2003	CCARM
NCCP_11439	ST5	2001	NCCP				

^1^ NT: Novel Type; indicating a sequence type not previously registered in the MLST database; NA: Not Available; indicating isolates where MLST alleles could not be fully typed; ^2^ ND: No data provided; ^3^ HIARC, Antibiotic Resistance Specialized Pathogen Resource Bank; NCCP, National Culture Collection for Pathogens; CCARM, Culture Collection of Antimicrobial-Resistant Microbes; Asan, Asan Medical Center.

**Table 2:** List of international reference MRSA genomes retrieved from the NCBI Sequence Read Archive (SRA).

Name	ST	Origin	Year	SRA No.
K10_1	ST1	Republic of Korea	2011	ERR10212939
K210_1	ST1	Republic of Korea	2012	ERR10212911
K110_1	ST188	Republic of Korea	2011	ERR10213024
K130_1	ST188	Republic of Korea	2012	ERR10213039
K170_1	ST188	Republic of Korea	2012	ERR10213077
K226_1	ST188	Republic of Korea	2011	ERR10212925
K60_1	ST188	Republic of Korea	2010	ERR10212982
K70_1	ST188	Republic of Korea	2010	ERR10212990
K190_1	ST20	Republic of Korea	2012	ERR10212891
s2004-ST239-1_1	ST239	China	2004	SRR30018423
s2010-ST239-6_1	ST239	China	2010	SRR30018353
s2015-ST239-22_1	ST239	China	2015	SRR30018336
ST239-20-85_1	ST239	China	2020	SRR30018323
K50_1	ST291	Republic of Korea	2010	ERR10212972
BD13502_1	ST5	USA	2011	SRR5909185
ISU931_1	ST5	USA	2010	SRR5909144
MN1_1	ST5	USA	2012	SRR5909263
MN4_1	ST5	USA	2013	SRR5909258
MN5_1	ST5	USA	2014	SRR5909259
MN50_1	ST5	USA	2012	SRR5909182
NRS49_1	ST5	China	1997	ERR2533592
s2004-ST5-5_1	ST5	China	2004	SRR30018318
s2010-ST5-1_1	ST5	China	2010	SRR30018293
s2015-ST5-21_1	ST5	China	2015	SRR30018405
ST5-20-25-118_1	ST5	China	2020	SRR30018385
UCI11_1	ST5	USA	2008	SRR5909280
UCI19_1	ST5	USA	2008	SRR5909253
UCI29_1	ST5	USA	2009	SRR5909232
UCI42_1	ST5	USA	2009	SRR5909174
UCI63_1	ST5	USA	2010	SRR5909163
K150_1	ST50	Republic of Korea	2012	ERR10213059
K30_1	ST513	Republic of Korea	2010	ERR10212956
KRSA10_1	ST5870	Republic of Korea	2018	SRR11788008
KRSA12_1	ST5870	Republic of Korea	2018	SRR11788006
KRSA9_1	ST5870	Republic of Korea	2018	SRR11788009
NCCP11854_1	ST59	Republic of Korea	2009	SRR30398400
K1_1	ST6	Republic of Korea	2011	ERR10212931
KRSA1_1	ST6	Republic of Korea	2018	SRR11787217
KRSA11_1	ST6	Republic of Korea	2018	SRR11788007
KRSA13_1	ST6	Republic of Korea	2018	SRR11788015
KRSA14_1	ST6	Republic of Korea	2018	SRR11788014
KRSA15_1	ST6	Republic of Korea	2018	SRR11788013
KRSA16_1	ST6	Republic of Korea	2018	SRR11788012
KRSA17_1	ST6	Republic of Korea	2018	SRR11788011
KRSA18_1	ST6	Republic of Korea	2018	SRR11788010
KRSA19_1	ST6	Republic of Korea	2018	SRR11788168
KRSA2_1	ST6	Republic of Korea	2018	SRR11787216
KRSA20_1	ST6	Republic of Korea	2018	SRR11788167
KRSA21_1	ST6	Republic of Korea	2018	SRR11788166
KRSA22_1	ST6	Republic of Korea	2018	SRR11788165
KRSA23_1	ST6	Republic of Korea	2018	SRR11788170
KRSA24_1	ST6	Republic of Korea	2018	SRR11788169
KRSA3_1	ST6	Republic of Korea	2018	SRR11787215
KRSA4_1	ST6	Republic of Korea	2018	SRR11787214
KRSA5_1	ST6	Republic of Korea	2018	SRR11787213
KRSA6_1	ST6	Republic of Korea	2018	SRR11787490
KRSA7_1	ST6	Republic of Korea	2018	SRR11787489
KRSA8_1	ST6	Republic of Korea	2018	SRR11787488
04-MR003_1	ST72	Republic of Korea	2004	SRR13039216
06-MS017_1	ST72	Republic of Korea	2006	SRR13039210
08-MR030_1	ST72	Republic of Korea	2008	SRR13039213
12-MS062_1	ST72	Republic of Korea	2012	SRR13039208
K07-204_1	ST72	Republic of Korea	2007	SRR11947553
K07-561_1	ST72	Republic of Korea	2007	SRR11948590
K100_1	ST72	Republic of Korea	2011	ERR10213014
K40_1	ST72	Republic of Korea	2010	ERR10212963
NCCP14750_1	ST72	Republic of Korea	2005	SRR26799573
ALE_SA_vanR_1	ST8	Republic of Korea	2020	SRR14868906
ALE_SA_vanR_2	ST8	Republic of Korea	2020	SRR14868907
ALE_SA_vanR_3	ST8	Republic of Korea	2020	SRR14868905
TCH1516_1	ST8	USA	2007	SRR6387495
NORSA1624_1	ST93	Australia	2013	SRR2057034
NORSA1626_1	ST93	Australia	2013	SRR2057035
NUM005_1	ST93	Australia	2013	SRR2057030
NUM043_1	ST93	Australia	2013	SRR2057031
NUM095_1	ST93	Australia	2013	SRR2057032
NUM096_1	ST93	Australia	2013	SRR2057033

## Data Availability

All tables, figures, and Supplementary Data presented in this article are available on the journal website. The raw data supporting the findings of this study are available from the corresponding author upon reasonable request.

## Supplementary Materials

The following supporting information can be downloaded at: https://www.mdpi.com/article/10.3390/antibiotics15030235/s1, Figure S1: Admixture CV value and Full plot; Figure S2: Profiling of virulence genes; Figure S3: IGV visualization to monitor ST239 recombination zone; Figure S4: CLC genomics workbench protocol. File S1: Supplementary command prompt.
